# The Role of Extracellular Vesicles in Cancer: Cargo, Function, and Therapeutic Implications

**DOI:** 10.3390/cells7080093

**Published:** 2018-08-01

**Authors:** James Jabalee, Rebecca Towle, Cathie Garnis

**Affiliations:** 1 Department of Integrative Oncology, British Columbia Cancer Research Center, Vancouver V5Z 1L3, BC, Canada; jjabalee@bccrc.ca (J.J.); rtowle@bccrc.ca (R.T.); 2 Division of Otolaryngology, Department of Surgery, University of British Columbia, Vancouver V6T 1Z4, BC, Canada

**Keywords:** extracellular vesicles, cancer, therapeutics

## Abstract

Extracellular vesicles (EVs) are a heterogeneous collection of membrane-bound structures that play key roles in intercellular communication. EVs are potent regulators of tumorigenesis and function largely via the shuttling of cargo molecules (RNA, DNA, protein, etc.) among cancer cells and the cells of the tumor stroma. EV-based crosstalk can promote proliferation, shape the tumor microenvironment, enhance metastasis, and allow tumor cells to evade immune destruction. In many cases these functions have been linked to the presence of specific cargo molecules. Herein we will review various types of EV cargo molecule and their functional impacts in the context of oncology.

## 1. Introduction

Extracellular vesicles (EVs) are a collection of lipid-bilayer enclosed vesicles secreted by virtually all cell types including cancer cells. EVs can be divided into subtypes based on their biogenesis, size and morphology, and collection method [1]. While other subtypes certainly exist, we use the term “EVs” to refer primarily to exosomes, ectosomes, and apoptotic bodies. Exosomes, the most heavily studied subtype, are ≈50–150 nm EVs formed by invagination of the multivesicular body (MVB) membrane. Once formed, exosomes are released into the extracellular space via fusion of the MVB with the plasma membrane. Ectosomes (sometimes called microvesicles or shedding microvesicles) are more heterogeneous, ranging in size from ≈100–1000 nm, and are formed through an outward blebbing of the plasma membrane. Apoptotic bodies are vesicles secreted by cells undergoing apoptotic cell death and range in size from ≈1000–5000 nm. Much of the work referenced herein is done on populations of EVs isolated via differential ultracentrifugation, which are likely enriched for exosomes compared to other subtypes. However, we use the term EVs to reflect the heterogeneous nature of these vesicles and the imperfect methods used to isolate them (which can lead to a mixture of EV subtypes). We occasionally use the term “exosomes” to refer to EVs pelleted by centrifugation at ≈100,000× *g*, “ectosomes” for EVs pelleted at ≈10,000 *g*, and apoptotic bodies for EVs pelleted at ≈2000 *g*. 

Although initially thought to function exclusively in the removal of unwanted molecules from cells, EVs are now recognized as important mediators of cell–cell communication. EVs play key roles in both normal and disease processes and are important regulators of cancer progression. EVs are known to contain cell-type specific cargo, including RNA, DNA, and protein, which are selectively sorted into EVs [2]. Once released, EVs can interact with cells in the immediate vicinity or at distant locations via transfer through the circulation. EVs interact with recipient cells in a number of ways, including ligand–receptor interaction [3], release of vesicle contents in the extracellular space by bursting [4], direct fusion with the plasma membrane [5], and endocytosis into the cell [6]. The latter mechanisms are of specific interest here as they result in a transfer of molecular cargo from EVs to the recipient cells [2,7]. In cancer, tumor cells both release and receive EVs. This crosstalk between tumor and stromal cells regulates numerous aspects of tumorigenesis, including growth of tumor vasculature [8], recruitment of cancer-associated fibroblasts [9], metastatic potential [8], and evasion of immune destruction [10]. Herein we describe each of the major cargo types associated with EVs, assess the functional impact of EVs on cancer biology, and address the potential clinical uses of EVs relating to their roles as biomarkers and therapeutics.

## 2. EV Cargo

### 2.1. EV Isolation and Cargo Profiling

The identification and accurate functional characterization of EV cargo requires appropriate isolation and purification methods. Currently, the most popular method of EV isolation remains differential ultracentrifugation (DU) [11]. DU involves removal of contaminating material through a series of low-speed centrifugations followed by pelleting of EVs at higher speeds. DU is a low-cost, high-throughput method, making it an ideal means of EV isolation in many labs. However, co-purification of non-vesicular proteins and other contaminants is an issue [12]. Combination of DU with other purification methods, such as ultrafiltration or density gradient centrifugation, can improve the purity of the collected vesicles at the expense of particle yield [12]. Furthermore, density gradient centrifugation can be laborious and is unable to separate EVs from contaminants of similar density. The use of alternative techniques, such as size-exclusion chromatography, immunoaffinity capture, microfluidics, and precipitation-based methods are on the rise, each with their own advantages and disadvantages that have been thoroughly examined elsewhere [13]. Briefly, size-exclusion chromatography results in excellent purity, but dilutes samples and therefore requires EVs be re-concentrated following isolation. In contrast, precipitation methods tend to result in high yield but relatively low purity. Immunoaffinity capture and microfluidics can be used to isolate EVs sharing a specific characteristic; for example, all EVs expressing CD63 on their surface [14]. However, markers capable of distinguishing between exosomes, ectosomes, apoptotic bodies, and other EVs have not yet been identified, and markers such as CD63 can be found on various EV subtypes [13]. It is worth noting that because these techniques isolate EVs according to different characteristics (e.g., size, density, presence of specific surface markers, etc.), they may enrich different vesicle subpopulations, potentially generating misleading results [15].

Once EVs have been isolated and purified, consideration must be given to how the cargo of interest (i.e., RNA, protein, DNA, etc.) is to be extracted and profiled. Highlighting the importance of extraction technique, EV RNA yield and size distribution were found to differ greatly depending on the method used, with column-based methods resulting in both the highest yield and broadest size range [16]. In some cases, additional treatment may be required to remove protein and nucleic acids from the outside surface of EVs. Indeed, the International Society of Extracellular Vesicles recommends investigators quantify EV RNA before and after vesicles are treated with proteinase/RNase to determine the contribution of surface-bound RNAs to the total RNA content collected [15]. For additional detail, we refer the reader to excellent recent reviews [13,15]. These studies serve to highlight the need for consistent and detailed reporting of experimental methods for the accurate interpretation of EV studies.

### 2.2. MicroRNA

Next-generation sequencing (NGS) of EV RNA cargo has revealed the presence of various classes of RNA in EVs derived from normal and cancer cells, including messenger RNA, transfer RNA, ribosomal RNA, microRNA, and more [17,18,19,20,21,22]. Among these, microRNAs (miRNAs), ≈22-nucleotide non-coding RNA molecules, are perhaps the most intriguing and heavily studied. A single miRNA species can regulate the translation and degradation of numerous mRNA targets, which makes miRNAs powerful regulators of cell phenotype. Although EV-mediated miRNA transfer has been strongly linked to cancer progression, the mechanisms underlying miRNA sorting into EVs are poorly understood.

MiRNA abundance in EVs is highly variable and depends on the cell line under study. MiRNAs were reported to comprise from 5–30% of EV small RNA content in colorectal and breast cancer cell lines [18,19], ≈50% in non-tumorigenic murine hepatocyte cells [23], and <1% in HEK293T cells [22]. The miRNA content of EVs is distinct from that of the parental cell, indicating that specific miRNA are selectively sorted into or excluded from EVs [7]. Although the mechanisms by which sorting occurs remain unclear, recent work has suggested that the recognition of specific miRNA motifs by RNA binding proteins (RBPs) may play a role. Specifically, the RBP hnRNPA2B1 was found to selectively sort miRNAs containing the GGAG motif in the 3′ half of their sequence [24], and the RBP synaptotagmin binding cytoplasmic RNA interacting protein (SYNCRIP) was found to selectively sort miRNAs containing the guanine-guanine-cytosine-uracil (GGCU) motif [23]. Furthermore, hnRNPA2B1 must be attached to small ubiquitin-like modifiers (SUMOylated) in order for sorting to occur, thus adding an additional layer of regulation [24]. How the RBP-miRNA complex is then selected for sorting remains an open question. In some cases, such as for hnRNPA2B1, the protein can be detected within EVs, suggesting the entire complex is sorted [24]. However, this may not be the case for all RBPs. Ubiquitination of the RBP HuR causes the protein to release its bound miRNA; ubiquitinated HuR was found to associate primarily with the MVB, where sorting into exosomes occurs, and had low affinity for its target miRNA compared to the non-ubiquitinated protein [25]. Interestingly, silencing of the RBP hnRNPH1 increased the total RNA in EVs, suggesting that RBPs may also exclude specific miRNAs from EVs [26]. In many cases, RBP-mediated changes in EV miRNA sorting have been linked to tumorigenesis. For example, major vault protein (MVP) regulates sorting of tumor suppressive miR-193a into EVs, effectively removing it from cells and leading to more aggressive disease [27]. The tumor suppressor VPS4A was found to have the opposite effect; in this case, overexpression of VPS4A was found to cause can accumulation of tumor suppressive miRNAs in cells and oncogenic miRNAs in their EVs, thus decreasing the growth, migration, and invasion of the cancer cells [28]. Additional examples of RBPs with known oncogenic or tumor suppressive functions that have been linked to EV miRNA sorting include Annexin A2 (ANXA2) [29], Kirsten rat sarcoma (KRAS) [18], Y-box binding protein 1 (YB-1) [22,30], MEX3C [31], and Argonaute 2 (AGO2) [32,33,34].

Interestingly, AGO2 is not the only component of the miRNA processing machinery found in EVs. The major components of the RNA-induced silencing complex (RISC)-loading complex machinery, including AGO2, Dicer, and trans-activation responsive RNA-binding protein (TRBP), were found in EVs of breast cancer cells where they actively processed pre-miRNA to mature miRNA, and Dicer sorting was found to be CD43-dependent [33]. In contrast, EVs of cultured monocytes were found to contain single-stranded, mature miRNAs but only low levels of AGO2 [35], suggesting that miRNA sorting occurs independently of RISC. Thus, two independent pathways, one involving the sorting of pre-miRNAs along with the RISC machinery and one involving the sorting of mature miRNAs, may exist [36].

In addition to RBPs, 3′-end nucleotide additions (NTAs) also regulate miRNA sorting. MiRNAs with 3′-end adenylation tend to be overrepresented in cells whereas those with 3′-end uridylation are overrepresented in EVs, particularly exosomes [37]. However, the underlying mechanisms remain unclear. It is unknown if uridylated miRNAs are specifically sorted into EVs and, if so, how sorting occurs. NTAs change the stability and activity of miRNAs, which may in turn affect their availability for sorting. Adenylation stabilizes miRNAs, allowing them to interact with their mRNA targets in the cell, whereas uridylation achieves the opposite [38]. The poor activity of uridylated miRNAs may decrease miRNA-mRNA interaction, allowing for the sorting of the free miRNA. Indeed, altering the expression level of a miRNA or its target mRNA can alter the quantity of that miRNA in EVs [39].

Finally, the biogenesis of EVs and the sorting of miRNA contents is regulated by the membrane lipid ceramide. In the case of exosomes, ceramide is produced via the breakdown of sphingomyelin by neutral sphingomyelinases (nSMases) at the MVB membrane. Inhibition of ceramide generation by the nSMase inhibitor GW4869 greatly reduces the small RNA content of EVs [40] and decreases the quantity of EVs released by cells in vitro [2]. Further, treatment of breast cancer cells with GW4869 decreased EV sorting of pro-angiogenic miR-210, thereby inhibiting tumor angiogenesis [8]. Ceramide is thought to play a role in sorting via the formation of ceramide-rich lipid microdomains. Sorting may occur directly via interaction of miRNA with ceramide-rich microdomains in the MVB membrane or indirectly via microdomain-dependent recruitment of proteins to the site of sorting [41]. Specific miRNA sequences show greater affinity for ceramide than others, thus providing a potential mechanism for sequence-based miRNA sorting [41]. Interestingly, ceramide appears to be required for miRNA sorting by some RBPs. Ceramide has been shown to co-localize with hnRNPA2B1 in the cytoplasm, and GW4869 inhibited HuR-mediated sorting of miR-122 and MEX3C-mediated sorting of miR-451a into exosomes [24,25,31]. Sorting of miR-451a was independent of the endosomal sorting complexes required for transport (ESCRT) pathway [31], discussed below.

EV miRNA content can also be modified by external factors, including hypoxia [42], carcinogens such as asbestos [43] or toluene [44], and infection with oncogenic viruses [45,46,47]. As with RBPs, these factors can alter EV miRNA content in ways that may promote or inhibit tumorigenesis. Viral infection provides an especially intriguing example of the power of EV miRNAs to promote tumorigenesis by influencing the tumor microenvironment. The genome of Kaposi’s sarcoma herpesvirus (KSHV) encodes a set of 12 latency-associated miRNAs, all of which have been found in the EVs of infected host cells [47]. Transfer of the viral miRNAs to non-infected cells via EVs results in a shift toward aerobic glycolysis which supports the growth of cancer cells by providing them with energy-rich metabolites [47]. In this way, virally-encoded EV miRNAs are used to reprogram the tumor microenvironment to enhance growth of KSHV-infected cancer cells. 

The sensitivity of EV miRNA content to genetic and environmental stimuli suggests their use as biomarkers. EV miRNA biomarkers show great promise; among their most exciting aspects are stability in the face of non-ideal collection methods, ability to be collected non-invasively, and specificity to certain disease states. With regard to non-invasive collection, potential EV miRNA biomarkers have been identified in numerous body fluids, including blood [43,48,49,50,51,52,53,54,55,56,57,58], urine [59,60,61,62], pleural effusion [63,64], and saliva [65]. Intriguingly, EV miRNA biomarkers appear exquisitely sensitive to specific disease states, and have been shown to discriminate among closely-related diseases, such as metastatic versus non-metastatic tumors [50], recurrent versus non-recurrent tumors [60], and high-grade versus low-grade tumors [56,60]. In addition, numerous studies have shown that EV miRNA biomarkers return to normal levels upon surgical resection of the tumor [51,56,61], suggesting their use in monitoring disease progression. While these results are encouraging, large-scale validation studies are required before EV miRNA biomarkers can be approved for clinical use. Along these lines, it is worth noting a few particularly promising biomarkers which have been found numerous times in separate studies. The oncomiR miR-21 has been proposed as a biomarker for various cancer types, including breast [49], prostate [59], bladder [61], brain [56], larynx [66], and liver [67]. MiR-375 [59,60,68] and the miR-200 family [21,51,63] show similar promise.

### 2.3. mRNA and Other RNA Types

Like miRNA, mRNA appears to make up a minority of EV RNA. In EVs derived from glioma stem-like cultures, mRNA accounted for <10% of the total RNA reads as assayed by NGS, and it is unclear what proportion of mRNA reads can be attributed to full-length mRNAs as opposed to mRNA fragments [18,69]. This may reflect a difference among EV subtypes, since mRNA appears to be more enriched in ectosomes compared to exosomes [69]. Despite uncertainty regarding mRNA abundance, numerous studies have shown that functional mRNAs can be transferred via EVs to recipient cells where they are translated and alter recipient cell phenotype [70,71,72,73]. For example, hTERT mRNA, which encodes the catalytic subunit of the telomerase enzyme, is transferred via EVs into nearby fibroblasts, thus increasing their proliferation, extending their life span, postponing senescence, and protecting from DNA damage [72]. Furthermore, hTERT mRNA was found in serum-derived EVs from 67.5% of 133 individuals with various cancers, but none of the 45 healthy controls [74], suggesting its use as a biomarker for detection of multiple cancer types. 

Recent studies suggest that the presence of specific sequence motifs play a key role in mRNA sorting into EVs. Interestingly, the YB-1 protein, which has also been linked to miRNA sorting [22,30], has been found to interact specifically with mRNAs whose 3′ untranslated region (UTR) contains any of three motifs, while the methyltransferase NSUN interacts with one motif [75,76]. YB-1 is overexpressed in numerous cancer types and drives cell proliferation [77], thus providing a link between cancer progression and EV packaging. Similarly, a 25-nucleotide motif was found to be enriched in the 3′ UTR of exosomal mRNAs from glioblastoma cells [78], whereas mRNAs harboring the signal peptide sequence were excluded from EVs [79]. 

Additional classes of RNA found in EVs include ribosomal RNA, transfer RNA, mitochondrial RNA, long non-coding RNA, piwi-interacting RNA, small nucleolar RNAs, and circular RNA [20,22,80,81,82]. Recent results have linked EV long non-coding RNAs to chemosensitivity [83] and cancer progression [84]. While outside the scope of the current manuscript, we refer the reader to an excellent recent review on this topic [85].

### 2.4. DNA

Extracellular vesicles carry DNA, which may be genomic (gDNA) [86,87] or mitochondrial (mtDNA) [88,89,90] in origin. Depending on the cell line and context, DNA may be single- or double-stranded, and may reside within the lumen or on the surface of EVs [88,91,92,93,94,95]. Surface-bound DNA can alter the ability of EVs to adhere to fibronectin [89], suggesting it may help determine how EVs interact with extracellular matrix molecules, such as those found in the tumor microenvironment or pre-metastatic niche. Luminal DNA, like other cargo types, can be transferred from donor to recipient cells resulting in increased mRNA and protein production, and oncogenes can be distributed among different cell types via this mechanism [92,94]. Intriguingly, EV-mediated spread of oncogenes has been shown to promote disease progression in mice. EVs derived from chronic myeloid leukemia (CML) cells transfer DNA encoding the breakpoint cluster region/Abelson murine leukemia viral oncogene homolog (BCR/ABL) fusion oncogene to the neutrophils of Sprague-Dawley rats and non-obese diabetic/severe combined immunodeficient (NOD/SCID) mice in vivo resulting in increased BCR/ABL mRNA and protein in the recipient murine cells and the eventual onset of CML-like characteristics [96]. Mitochondrial DNA in EVs also appears to be functional in recipient cells, and cancer-associated fibroblast-derived mtDNA has been found to play a role in the resistance of breast cancer cells to hormone therapy [90]. 

While current work has shed light on the functions of EV DNA, little is known regarding how it is sorted into EVs. Numerous studies have reported that cancer cell-derived EVs contain gDNA from all chromosomes [86,93,95,97], and at least one study has provided evidence for the packaging of the entire mitochondrial genome within EVs [90]. These results suggest that selective sorting of specific DNA sequences may not occur. Interestingly, knockdown of EV release in human fibroblasts and various cancer cell lines results in the accumulation of damaged DNA in the cytoplasm [98]. Such cytoplasmic DNA can be recognized by DNA sensing proteins, the activation of which results in genomic DNA damage, senescence, or apoptosis [98]. Considering this evidence, it is possible that EVs play a role in maintaining cellular homeostasis through non-selective removal of cytoplasmic DNA [98]. In contrast to the idea of non-specific gDNA packaging, apoptotic bodies, ectosomes, and exosomes were found to contain shared and unique DNA sequences [86], suggesting that independent DNA packaging mechanisms for each of the vesicle subtypes may exist. More work is required to clarify the mechanisms underlying EV DNA packaging and its functional relevance to normal and cancer cells.

Intriguingly, cells with specific mutations in their gDNA release EVs containing DNA that harbor identical mutations. Indeed, EV DNA containing mutations identical to the gDNA has been found in cell culture supernatants [86,93,95], the plasma of tumor-bearing mice [95], and the blood (serum and plasma) of human cancer patients [93,97,99,100,101,102] (Table 1). Blood-derived EV DNA provides a rich source of clinically relevant information. NGS was used to detect at least 10 potentially clinically actionable mutations in the EV DNA of patients with pancreatic cancer [97], and a similar approach was used to detect specific mutations in three well-known oncogenes in patients with different cancer types with an overall sensitivity of 95% [100]. A major advantage of mutational analysis of EV DNA compared to other techniques, such as standard biopsies, is the ability to collect samples throughout the course of treatment, thus decreasing the need for invasive biopsies. This flexibility allows physicians to monitor genomic changes in the tumor over time.

Interestingly, recent reports suggest that cell-free circulating tumor DNA (cfDNA), which was previously thought to derive primarily from apoptotic and necrotic tumor cells, is comprised largely of EV DNA [103]. Many companies offer panels for the comprehensive detection of cfDNA mutations in cancer-related genes, and analysis of mutations in cfDNA is currently being used to guide patient management [104]. By simultaneously collecting and analyzing cfDNA, exosomal DNA, and exosomal RNA from the serum of non-small cell lung cancer patients, Castellanos-Rizaldo and colleagues detected the EGFR T790M mutation with 92% specificity and 89% sensitivity when compared to tissue biopsy [102]. These results highlight the exciting clinical applications of EV DNA. A deeper understanding of EV biology-such as how specific molecular targets are selected for packaging and the identification of markers for the separation of tumor and non-tumor-derived EVs-will serve to further refine the utility of EV-based biomarkers in the clinic.

### 2.5. Protein

In addition to being transferred to recipient cells and influencing cell phenotype, proteins also regulate the sorting of other EV components, determine which cell types are able to receive EVs (i.e., determine tropism), provide markers for the separation of EV subtypes, bind to and activate receptors on recipient cells, and carry out cell-independent reactions inside of EVs after their release. Here we will discuss the sorting of specific proteins followed by a brief discussion of a few of their various other functions in relation to cancer biology. 

A key pathway of EV protein sorting involves the endosomal sorting complexes required for transport (ESCRT), a series of four protein complexes (ESCRT-0, I, II, and III), and accessory proteins. ESCRTs recognize and bind ubiquitinated proteins, facilitating their sorting into EVs [105]. In addition to ubiquitination, other post-translation modifications appear to play an important role in EV protein sorting through both ESCRT-dependent and ESCRT-independent pathways, at least for some proteins. Phosphorylation may either promote or inhibit EV sorting, as evidenced by EPHA2 and AGO2, respectively [34,106]. As mentioned above, SUMOylation of the RBP hnRNPA2B1 regulates sorting into exosomes [24]. Other mechanisms of protein sorting into EVs involve dimerization [107], and recruitment via other proteins, such as tetraspanins [108]. 

The oncogenic activity of EVs is dependent not only upon their intraluminal cargo, but also on the array of proteins that span the EV membrane. One intriguing function of such transmembrane proteins involves determining EV tropism; i.e., which cell types are most likely to take up, and thus be influenced by, EVs. EV tropism has been observed in vitro among different cell types. In vivo, EVs from metastatic cell lines are more likely to be taken up by resident cells at sites to which those lines commonly metastasize [109,110]. Especially important in this process are integrins (ITGs), a class of proteins known to facilitate cell-extracellular matrix interactions. ITGα_6_, and its partners ITGβ_4_ and ITGβ_1_, are abundant in EVs that distribute mainly to the lung where they are taken up primarily by S100A4-positive fibroblasts, ITGβ_5_ and ITGα_V_ are abundant in EVs that distribute mainly to the liver where they are taken up primarily by Kupffer cells, and ITGβ_3_ is abundant in EVs that distribute mainly to the brain where they are primarily taken up by endothelial cells [110]. The uptake of EVs at specific locations within the body appears to play a key role in determining the location of metasteses, as evidenced by the observation that injection of lung-tropic EVs into mice increased the lung metastatic capacity of breast cancer cells which normally metastasize preferentially to bone [110]. Moreover, EVs can alter gene expression in recipient cells of the pre-metastatic niche, which may include cancer and stromal cells [109,110,111,112]. For example, astrocyte-derived EV miRNAs inhibit the tumor suppressor PTEN in cells that metastasize to the brain, thus priming them for metastatic outgrowth [111]. In addition, miR-122-containing breast cancer cell-derived EVs prime the premetastatic niche by decreasing expression of the glucose metabolizing enzyme pyruvate kinase in nearby stromal cells, thus increasing nutrient availability for metastasizing cancer cells [112]. In contrast to integrins, which direct EVs to their preferred targets, additional proteins have been identified which function to modify or block EV uptake. Along these lines, REG3β interferes with the uptake of EVs into target cells by binding to glycoproteins on the EV surface [113]. Similarly, CD47, an anti-phagocytic signal, was found to block uptake of EVs by immune cells, thus prolonging their time in circulation [114]. These results suggest that manipulation of the surface proteins of EVs could be used to alter their tropism and block their pro-tumor effects.

In addition to tropism-determining proteins, the presence of which is dependent upon cell type, other proteins have been shown to be more ubiquitously found in EVs and can provide information on their cellular origin. In an intriguing example, immunoaffinity capture was used to separate A33-positive and EpCAM-positive exosomes secreted from colorectal cancer organoids, and each population was found to be enriched for distinct proteins [115]. These results suggest that even within the EV subpopulation of exosomes, additional subpopulations, which may differ in aspects of their biogenesis and cargo, are likely to exist, a result supported by others [116]. Tetraspanins, a family of membrane-spanning proteins which includes CD63, CD9, and CD81, are among the most commonly cited “exosome markers”. Unfortunately, the assumption that such markers are found on all exosomes is an over-simplification; truly specific markers that are found on all exosomes do not exist. CD63 and CD9 were also found, to differing degrees, to be present on other EV subtypes including ectosomes and apoptotic bodies [116], and particles isolated by ultracentrifugation (which are assumed to be enriched for exosomes) can be separated into CD63+, CD9+, CD81+, and non-tetraspanin-bearing subpopulations, although overlap between markers on a single EV is common [116]. The distinction among exosome subtypes may be an important one due to the many key roles tetraspanins play in EV biology, including regulation of biogenesis [117], cargo sorting [117], and tropism [118]. Thus, subpopulations of exosomes that differ in their tetraspanin content may also differ in their biological function. Further detailed investigation into how best to obtain and purify EVs based on protein markers, and whether such distinctions are truly biologically relevant, is warranted. 

The separation of EV subtypes based on surface proteins may prove clinically useful. By studying the surface proteins of EVs collected from cancer and non-cancer cell lines, Melo and colleagues found dozens of cancer cell-specific markers [119]. One of these, GPC1, was found to increase in the blood of patients with breast and pancreatic cancer compared to controls, suggesting its use as a disease detection biomarker [119]. Using similar methodology, Castillo and colleagues identified proteins specific to the EVs of pancreatic cancer cells but not normal controls [120]. Unfortunately, GPC1 was not found in pancreatic cancer EVs, and appeared instead to be selectively expressed in non-cancerous tissues [120]. Once refined, separation of cancer and normal EVs will increase the yield of tumor-specific material and decrease unwanted background in downstream analyses.

## 3. Extracellular Vesicle Function in Cancer

### 3.1. Impact of EVs on Fibroblasts

**Figure 1:**
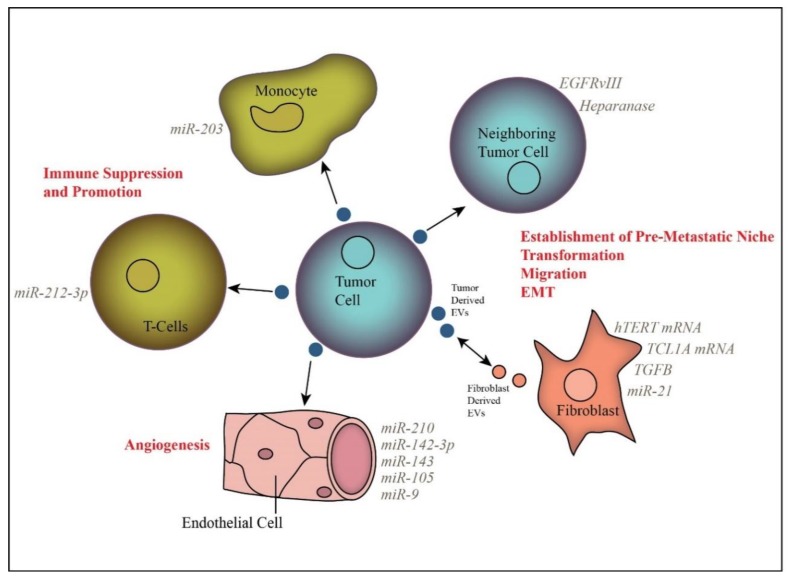
Extracellular vesicle-mediated transfer of specific cargo molecules alters the phenotype of recipient cells, including neighboring tumor cells, fibroblasts, endothelial cells, and immune cells.

Release of EVs by tumor cells is believed to play a major role in intercellular communication, facilitating signaling to surrounding tumor cells and to distant sites via blood or other biological fluid transportation (Figure 1). A primary focus of inquiry has been the impact of EVs on the tumor stroma, including fibroblasts, endothelial cells, and immune cells. 

Fibroblasts comprise a major component of the tumor stroma. Under tumorigenic conditions, fibroblasts can undergo morphological changes that confer a phenotype similar to myofibroblasts, which are activated, mobile fibroblasts. Interestingly, tumor-derived EVs are able to induce the transformation of normal stromal fibroblasts into activated cancer-associated fibroblasts (CAFs) [121,122,123,124,125]. For instance, TGFβ-containing prostate cancer-derived EVs are sufficient to induce fibroblast transformation to a CAF-like phenotype [123]. Further, the resultant increases in wound healing and endothelial cell growth were more pronounced in EV-exposed fibroblasts as compared to fibroblasts transformed by soluble TGFβ alone [123]. The ability of EVs to promote CAF activation was found to correlate with the aggressiveness of the tumor cells, with EVs from a more aggressive cell line prompting higher CAF marker expression, proliferation rate, and enzyme release by treated fibroblasts than EVs from a less aggressive cell line [126].

Once activated, CAFs secrete EVs that promote tumorigenesis by increasing proliferation, motility, epithelial-mesenchymal transition, migration, and metabolic changes in tumor, endothelial cells, and other fibroblasts [126,127,128,129]. Especially intriguing is the role of EVs in mediating resistance to chemotherapy [127,130]. For instance, exposing pancreatic ductal carcinoma cells to conditioned pancreatic fibroblast media was sufficient to confer resistance to gemcitabine, potentially due to up-regulation of snail family transcriptional repressor 1 (SNAIL) and miR-146a in the recipient cells [127]. Gemcitabine treatment also led to an increase in CAF EV secretion, indicating a potential mechanism of drug resistance in pancreatic cancer [127].

### 3.2. EVs Induce Angiogenesis in Endothelial Cells

The ability to induce angiogenesis is a hallmark of cancer, and recent evidence suggests that EVs are key regulators of tumor vascularization via transfer of pro-angiogenic molecules from tumor to endothelial cells. Indeed, EVs have been shown to increase tube formation, migration, cell–cell adhesion, and proliferation in endothelial cells in a variety of cancer types [8,122,131,132,133,134,135,136,137,138]. For example, activated EGFR found in EVs is sufficient to induce EGFR and VEGFR signaling in recipient endothelial cells, and blocking EV-mediated EGFR transfer decreased tumor growth and angiogenesis [131,139]. Furthermore, EVs produced by hypoxic tumor cells have been shown to have a more pronounced effect on endothelial cells in promoting angiogenesis than those derived from normoxic cells [134,137]. Hypoxia increases the production of tumor and stromal cell-derived EVs and alters their cargo [42,137,140,141,142]. For example, miR-23a is found in the EVs of hypoxic, but not normoxic, lung cancer cells and promotes angiogenesis through the inhibition of prolyl hydroxylase in recipient endothelial cells [141]. Increased EV production by hypoxic endothelial cells was abrogated by siRNA targeting hypoxia inducible factor 1α, thus providing a clear link between cell response to hypoxia and EV production [142]. Other EV-derived molecules that have been shown to play a role in promoting angiogenesis include miR-9, miR-105, miR-142-3p, miR-210, and H19 lncRNA [132,133,135,138,141,143,144].

Several groups have looked at the impact of sub-populations with cell markers indicative of tumor initiating cells. In renal cell carcinoma cell lines, CD105-positive cells were found to release EVs that increase proliferation, vessel formation, and invasion in HUVEC endothelial cells, whereas CD105-negative cells did not [134]. Similarly, in liver cancer cells, CD90-positive cells were found to secrete EVs that promote tube formation and cell–cell adhesion via transfer of H19 lncRNA [132]. These results highlight the heterogeneity found within tumors and suggest that subsets of tumor cells secrete EVs carrying a unique set of cargo capable of altering stromal cell phenotypes in specific ways.

### 3.3. Extracellular Vesicles in Immunomodulation

EVs are an important mode of communication among cells of the immune system and are key regulators of the anti-cancer immune response. Initial reports showed that EVs secreted by dendritic cells induce an antitumor immune response, suggesting the use of immune cell-derived EVs as an anti-cancer vaccine as discussed below [145]. This work was strengthened by the observation that EVs contain proteins involved in antigen presentation and immune stimulation, including tumor antigens and major histocompatibility complex (MHC) proteins [146,147]. However, additional work indicated that tumor-derived EVs often promote immune suppression. 

Among the most heavily studied immune cell recipients of tumor-derived EVs are dendritic cells and T cells. In many instances, tumor-derived EVs have been found to have an inhibitory effect on dendritic cell function [146,148]. For instance, pancreatic cancer-derived EVs containing miR-203 were found to impair dendritic cell function via reduction of toll-like receptor 4 expression [146]. Furthermore, pancreatic cancer cell-derived EVs were found to alter the transcriptional profile of recipient dendritic cells via transfer of miRNA, specifically miR-212-3p [148]. This led to a decrease in MHCII expression and suppressed immune function. There are conflicting reports on the role of tumor-derived EVs in dendritic cell maturation, with different studies citing either inhibitory or stimulatory effects [149,150,151,152].

EVs can also impact T-cell function either directly or via inhibition of other immune cell types, such as dendritic cells [152]. Several studies found that tumor-derived EVs can affect T-cell function, specifically by increasing proliferation, differentiation, and induction of T regulatory cells that function to blunt the immune response [151,153]. Interestingly, direct inhibition of T cell function via EV-associated PD-L1 has also been reported [154]. Furthermore, prostate cancer cell-derived EVs have been found to down-regulate NKG2 in natural T-killer cells and this could contribute to immune suppression [150]. These examples serve to underscore the variety of ways in which tumor-derived EVs can inhibit the immune system.

EVs may also act on cells of the immune system to promote tumor-supportive inflammation. For example, tumor cell-derived EVs can stimulate macrophages to release pro-inflammatory cytokines via activation of the NFκB pathway [155,156]. In breast cancer cells, this pathway activation led to an increase in the secretion of pro-inflammatory cytokines including IL-6, TNFα, GCSF, and CCL2 [156]. Furthermore, tumor EV-associated miR-21 and miR-29a can trigger a pro-inflammatory response in immune cells [157]. Interestingly, these miRs appear to function by acting as EV-associated ligands for toll-like receptors, rather than through their internalization into the cell [157].

### 3.4. Tumor Promoting Effects of Other Extracellular Vesicles

As noted above, the majority of studies have focused on the functional impact of EV populations collected via ultracentrifugation at 100,000× *g*, which are assumed to be enriched for exosomes. Several papers, however, have also assessed larger EV species, including ectosomes and large oncosomes, which arise from non-apoptotic blebbing of the plasma membrane [124,158,159]. This subset of EVs is obtained by collecting pellets from cell culture media supernatant centrifuged at 10,000× *g* and further purified by density gradient centrifugation. Minciacchi and colleagues found that large oncosomes were able to reprogram prostate fibroblasts via alterations in MYC/AKT1 pathways, rather than via TGFβ as was observed by exosomes [123,158]. Thus, different subtypes of EV reprogram fibroblasts using different mechanisms. Interestingly, by comparing the tumorigenic capabilities of the 10,000 *g* and 100,000 *g* EV fractions from the same cell line, Lindoso and colleagues found that EVs collected after centrifugation at 10,000× *g* were more effective at stimulating angiogenesis, whereas EVs collected after centrifugation at 100,000× *g* were more effective at increasing migration of endothelial cells [124]. These results strengthen the conclusion that different EV subtypes perform unique functions within the tumor niche.

## 4. Therapeutic Implications of Extracellular Vesicles

In addition to EV biomarkers derived from serum or other biological fluids, a topic that has been thoroughly reviewed above and by others [160,161], EVs have significant potential for use in anti-cancer therapy. Strategies include using EVs as potential cancer vaccines or drug delivery systems, developing interventions to sequester tumor-derived EVs in patients, and developing drugs that target factors involved in EV release.

One of the first indications that EVs may have utility as cancer therapeutics was the observation that dendritic cells secrete antigen-presenting vesicles and that tumor peptide-pulsed dendritic cell-derived EVs decrease tumor growth in mice [145]. This finding drove interest in using dendritic cell-derived EVs as tumor vaccines and spurred multiple clinical trials. Three Phase I trials confirmed the safety of use of dendritic cell-derived EVs in anti-cancer treatments; however, the injected EVs exhibited poor potential in stimulating a T-cell response in the patients [162,163,164]. More recently, a Phase II trial was completed using dendritic cell-derived EVs as a vaccine. This involved EVs derived from IFN-γ-matured dendritic cells rather than immature dendritic cells [165]. Unfortunately, the endpoint goal (4 months of disease-free survival in 50% of patients) was not reached. A major hurdle in the use of EVs as therapeutics involves the standardization of techniques used to collect and analyze EVs and their molecular cargo, as discussed in Section 2.1 [13]. Interestingly, Tkach and colleagues found that EVs derived from immature dendritic cells are functionally heterogeneous, with large (2000 *g*) and small (100,000 *g*) EVs resulting in different cytokine expression profiles in recipient cells [166]. However, no such heterogeneity was observed for EVs derived from mature dendritic cells [166]. While promising, further work is required to develop a suitable strategy for use of dendritic cell-derived EVs as a form of anti-cancer therapy. 

EVs display characteristics that make them ideal options for drug delivery. They are well tolerated in the body, easily taken up by cells, and can be targeted for uptake by specific tissues [167]. A general strategy involves engineering EVs to contain a specific cargo, such as pro-apoptotic proteins, miRNAs, or siRNAs, chemotherapeutic drugs, or molecules targeting specific oncogenes [114,168,169,170,171,172,173]. Indeed, several papers have described the successful insertion of siRNAs into exosomes [114,169,170,171]. For example, Alvarez-Erviti and colleagues successfully used self-derived dendritic cell EVs loaded with siRNA to target the brains of mice, finding that this approach did indeed facilitate knockdown of target mRNAs [169]. Further, injection of mice with EVs engineered to contain siRNA targeting mutant KRAS suppressed pancreatic cancer growth and improved overall survival [114].

An additional approach involves counteracting the pro-tumorigenic effects of EVs. One approach is to directly remove EVs from circulation. For example, Marleau and colleagues describe an extracorporeal hemofiltration system which filters blood for components under 200 nm and removes them using affinity agents for target molecules [174]. However, additional studies are required to test the clinical utility of this device. As another example, Nishida-Aoki and colleagues found that treatment of mice with anti-CD9 or anti-CD63 antibodies stimulated EV removal by macrophages, thus greatly decreasing EV concentration in the blood [118]. Although this treatment had no effect on the primary tumor, the authors observed a significant reduction in metastasis [118]. Blocking EV biogenesis in tumor cells by silencing genes encoding EV-related machinery is another potential avenue for inhibiting tumorigenesis. For example, knockdown of *SMPD3* and *RAB27A* resulted in reduced EV secretion and decreased tumorigenesis in mouse models [127,133,143]. However, such strategies may interfere with the normal process of EV-mediated communication; thus, a strategy which serves to minimize off-target effects is required.

## 5. Summary

In the past few years we have learned a great deal regarding the myriad of cargo molecules contained within EVs and the complex roles EVs play in the tumor microenvironment. The pace of research on this topic has vastly increased in the past couple of years, and we will no doubt make great strides in the years ahead in understanding the complexities underlying the role of EVs in cancer. Though much of this research is still in its infancy, there no doubt there lies many exciting therapeutic and biomarker opportunities ahead.

## Figures and Tables

**Table 1:** Summary of recent publications using EV DNA for detection of specific mutations in cancer-related genes.

Study	Fluid	Cancer Type	Patients	Technique	Genes Analyzed	Results
Kahlert et al., 2014 [93]	Blood (serum)	Pancreatic ductal adenocarcinoma	Human; 2 cancer (no stage given), 2 healthy	PCR, Sequencing (BigDye terminator kit)	KRAS, TP53	Detected two different KRAS mutations and one TP53 mutation.
Lázaro-Ibáñez et al., 2014 [86]	Blood (plasma)	Prostate	Human; 4 cancer (T stages 1–3), 4 healthy	PCR, sequencing (BigDye terminator kit)	MLH1, PTEN, TP53	Unable to detect specific mutations.
Thakur et al., 2014 [95]	Blood (plasma)	Melanoma	SK-MEL-28 cells xenografted into NOD/SCID mice; EVs collected when tumors reached max allowable size	Allele-specific PCR	BRAF (V600E)	Mutation detected.
San Lucas et al., 2016 [97]	Blood and pleural fluid	Pancreatic ductal adenocarcinoma (PDAC) and ampullary adenocarcinoma	Human; 2 PDAC and 1 ampullary adenocarcinoma	Next-generation sequencing	Whole genome	At least 10 potentially clinically actionable mutations identified in each patient.
Allenson et al., 2017 [99]	Blood (plasma)	Pancreatic ductal adenocarcinoma (PDAC)	Human; 68 PDAC (all stages), 20 PDAC patients whose blood was drawn after resection with curative intent, and 54 healthy controls	Droplet digital PCR	KRAS	Mutations detected in 7.4%, 66.7%, 80%, and 85% of controls, localized, locally advanced, and metastatic PDAC patients.
Möhrmann et al., 2017 [100]	Blood (plasma)	46.5% colorectal, 18.6% melanoma, 14.0% non-small cell lung cancer, 20.9% other	Human; 43 progressing advanced cancers	Next-generation sequencing	BRAF^V600^, KRAS^G12/G13^, EGFR^exon19delL858R^	Mutations in EV DNA which correspond to those in tissue found in 95% of cases. EV DNA did not contain mutations not present in the parental tumor cells.
Yang et al., 2017 [101]	Blood (serum)	Pancreatic ductal adenocarcinoma (PDAC), chronic pancreatitis (CP), intraductal papillary mucinous neoplasm (IPMN)	Human; 48 PDAC, 9 CP, 7 IPMN, 114 healthy controls	Digital PCR	KRAS^G12D^, TP53^R273H^	KRAS mutation detected in 39.6% PDAC, 28.6% IPMN, 55.6% CP, 2.6% healthy controls. TP53 mutation detected in 4.2% PDAC, 14.2% IPMN, 0% CP, 0% healthy controls.
Castellanos-Rizaldos et al., 2018 [102]	Blood (serum)	Non-small cell lung cancer	Human; Training and test cohorts each with 51 mutation positive and 54 mutation negative samples	Allele-specific PCR	EGFR^T790M^	Training: 81% sensitivity, 95% specificity. Test: 92% sensitivity, 89% specificity
